# Prevalence and risk factors of depression in Chinese patients with type 2 diabetes mellitus: a protocol of systematic review and meta-analysis

**DOI:** 10.1186/s13643-021-01855-7

**Published:** 2021-11-25

**Authors:** Xiaobo Liu, Chao Dong, Hong Jiang, Dongling Zhong, Yuxi Li, Huiling Zhang, Jun Zhang, Jin Fan, Juan Li, Li Guan, Rongjiang Jin

**Affiliations:** 1 School of Health Preservation and Rehabilitation, Chengdu University of Traditional Chinese Medicine, Chengdu, China; 2 The General Hospital of Western Theater Command, Chengdu, China; 3 Department of Rehabilitation, Xichong County People’s Hospital, Nanchong, China; 4 School of Acupuncture-Moxibustion and Tuina, Chengdu University of Traditional Chinese Medicine, Chengdu, China; 5 Department of Rehabilitation, Fushun County People’s Hospital, Zigong, China

**Keywords:** Type 2 diabetes mellitus, Depression, Prevalence, Risk factors, China

## Abstract

**Background:**

The prevalence of type 2 diabetes mellitus (T2DM) is growing in China. Depression is a significant complication of T2DM, leading to poor management of T2DM. Thus, early detection and treatment of depression in patients with T2DM are essential and effective. Therefore, we plan to conduct a systematic review and meta-analysis to evaluate the prevalence of depression in Chinese patients with T2DM and explore potential risk factors of depression in T2DM.

**Methods:**

We will search literatures recorded in MEDLINE, EMBASE, the Cochrane Library, Chinese Biomedical Literature Database (CBM), China National Knowledge Infrastructure (CNKI), Chinese Science and Technology Periodical Database (VIP), and WanFang Database from their inception onwards. We will manually search gray literatures, reference lists of identified studies, relevant websites, and consult experts in this field. We will include population-based, cross-sectional surveys that investigated the prevalence of depression in Chinese patients with T2DM or/and the possible risk factors of depression in T2DM. Two reviewers will screen studies, extract data, and evaluate risk of bias independently. Agency for Healthcare Research and Quality methodology checklist will be used to assess the risk of bias. If feasible, we will conduct random effects meta-analysis of observational data to summarize the pooled prevalence, and use odds ratio for categorical data to explore potential risk factors. Prevalence estimates will be stratified according to age, gender, and other factors. Statistical heterogeneity will be estimated using Cochran’s *Q* and *I*^*2*^ index. We will conduct meta-regression to investigate the potential sources of heterogeneity, sensitivity analyses to assess robustness of the synthesized results, and funnel plots and Egger’s test to assess publication bias.

**Discussion:**

This systematic review and meta-analysis will provide comprehensive evidence of the prevalence and potential risk factors of depression in Chinese patients with T2DM. We expect to provide evidence for healthcare practitioners and policy makers to pay attention to the mental health of patients with T2DM. Our data will highlight the need and importance of early detection and intervention for depression in patients with T2DM.

**Systematic review registration:**

PROSPERO CRD42020182979.

**Supplementary Information:**

The online version contains supplementary material available at 10.1186/s13643-021-01855-7.

## Background

Type 2 diabetes mellitus (T2DM) is a common chronic and metabolic disease, accounting for between 90 and 95% of all diabetes [1]. Globally, low- and middle- income countries have the highest proportion of T2DM [2]. According to international diabetes federation (IDF) [3] and summary data by Ma RCW [4], with sharp diabetic prevalence rise, China has the highest diabetes populations around the world, which mainly attributed to T2DM [4]. The latest statistics showed that the overall prevalence of T2DM in China was 9.1% [5].

With the progress of disease, patients with T2DM are likely to develop psychological comorbidities, such as depression [6, 7]. Depression is characterized by persistent sadness and a loss of interest or pleasure in previously rewarding or enjoyable activities [8]. Plenty of studies reported a bi-directional relationship between T2DM and depression [9], which indicates that they are risk factors to each other [10–12]. Study reported that people with T2DM had a 24% increased risk to develop depression compared to the healthy based on global data [13]. This is concerning since comorbid depression in T2DM means poorer compliance [14] and worse quality of life [15], higher risk of dementia [16], and cardiovascular event [17, 18]. In view of large T2DM population in China and bad influence of depressive symptoms on patients with T2DM, knowing the current prevalence of depression in Chinese patients with T2DM and identifying high-risk groups are meaningful and urgent.

Epidemiological results from several developing countries showed the prevalence of depression in T2DM ranged from 34 to 54% [19–21]. Based on global statistics, Khaledi M et al. reported 28% T2DM patients had depression at different degrees or major depressive disorder [22], and Wang F et al. found 14.5% T2DM were diagnosed as major depressive disorder [23]. In China, relevant epidemiological investigations have been carried out in quantity. Numerous cross-sectional surveys of prevalence of depression in Chinese T2DM patients have been conducted [24, 25]. Meanwhile, several studies have reported various risk factors associated with the depression in T2DM patients, such as gender, age, educational level, complications, and lifestyles (e.g., smoking, drinking, and exercise). However, some of the results were inconsistent [25–29]. A systematic review (SR) of prevalence of depression in Chinese T2DM patients conducted by Wang B et al. [30] included 28 studies published up to 2016. Since more surveys have been published in the recent years, we plan to conduct a SR and meta-analysis based on cross-sectional study to evaluate the up-to-date prevalence and potential risk factors of depression among adult patients with T2DM in China.

### Research questions

This SR and meta-analysis will answer the following research questions:

1. What is the prevalence of depression among adult patients with T2DM in China?
2. What are the potential risk factors associated with the depression in T2DM?

## Methods/design

### Study registration

This SR protocol has been priori registered on PROSPERO at 30 April 2020 (registration ID: CRD42020182979). Our protocol is reported in according to the Preferred Reporting Item for Systematic Review and Meta-analysis-Protocol (PRISMA-P) statement [31] (Additional file 1) and referred to the MOOSE guidelines for Meta-Analyses and Systematic Reviews of Observational Studies [32]. The results of this SR will be reported according to the Preferred Reporting Items for Systematic review and Meta-Analysis (PRISMA) 2020 statement [33] and MOOSE guidelines.

### Inclusion criteria

We will include studies based on the following criteria:

#### Studies characteristics

1. Population-based, cross-sectional surveys investigated the prevalence of depression in Chinese patients with T2DM or/and researched the potential risk factors associated with depression in T2DM, regardless of sample size
2. Studies published in English or Chinese

#### Participant characteristics

1. Chinese adults (≥ 18-year old) were diagnosed with T2DM based on self-reported physician’s diagnosis, medical records or glucose level testing (fasting plasma glucose ≥7.0 mmol/L and/or 2 h plasma glucose ≥11.1 mmol/L) [34]
2. T2DM patients were identified as depression by screening instruments which have been verified to have good validity and reliability in Chinese population (e.g., Patient Health Questionnaire-9, Hamilton Depression Scale, Self-rating Depression Scale, Center for Epidemiologic Studies Depression Scale, Beck depression inventory, Composite International Diagnostic Interview, the Geriatric depression) [35], or patients were diagnosed as major depressive disorder by operationalized criteria such as the Diagnostic and Statistical Manual of Mental Disorders (DSM) or the International Statistical Classification of Diseases and Related Health Problems 10th Revision (ICD-10)
3. Participants living in Chinese regions, and no restrictions on gender

#### Type of outcome measurements

1. Our primary outcome will be the pooled prevalence of depression in Chinese patients with T2DM.
2. The secondary outcome will contain the potential risk factors of depression in patients with T2DM in China, such as gender, age, educational level, complications, and lifestyles (e.g., smoking, drinking, and exercise).

### Exclusion criteria

We will exclude studies meeting one of the following criteria:

1. Hospital-based studies
2. Randomized trials, case studies, qualitative studies, systematic reviews, protocols, commentaries, editorials, and conference abstracts
3. Full text or data are unavailable by any useful approach
4. Repeated published studies

### Databases and search strategy

Electronic databases including MEDLINE, EMBASE, the Cochrane Library, Chinese Biomedical Literature Database (CBM), China National Knowledge Infrastructure (CNKI), Chinese Science and Technology Periodical Database (VIP), and WanFang Database will be searched from inception onwards. Both Medical Subject Headings (MeSH) and free-text words related to China, diabetes and depression will be used for searching relevant articles. The search strategy of MEDLINE was shown in Additional file 2. Suitable strategies will be applied in other electronic databases in accordance with respective retrieval rules. We will search reference lists of included articles and relevant SRs for additional eligible studies. Gray literature will also be manually searched such as conference proceedings and academic degree dissertations. Relevant websites will be searched for useful data, and experts in this field will be consulted for possible relevant studies.

### Study selection

We will use reference management software (ENDNOTE X9) to manage the all articles collected from literature search. After filtering out the duplicate records, two independent reviewers (HLZ and JZ) will screen the titles and abstracts of the rest records to acquire potentially eligible studies preliminary. Then, full texts will be obtained and checked independently by two reviewers (XBL and JF) to identify the eligible studies. All screening processes will be based on inclusion and exclusion criteria. Any disagreements will be discussed with a third reviewer (JL) to meet a consensus. We will provide a list of excluded studies and justify the exclusions. PRISMA 2020 flow diagram will be presented to describe the screening and selection processes [33].

### Data extraction

Once eligible studies are identified, two reviewers (YXL and DLZ) will independently extract the data using a prepared data extraction form. The extracted data from each study will include the following:

1. Study characteristics: title, journal, conducted year(s), country, geographical region, method of data collection, criteria used to identify diabetes and depression, source of funding
2. Participants’ information: mean age, gender, history of diabetes, education level, lifestyles (e.g., smoking, drinking, and exercise), diabetes treatment (e.g., diet control, oral medicine, use of insulin), and other relevant reported demographic, health, and diabetes-related information
3. Critical data: sample size, the number of subjects with depression, response rate, the reason for non-response, potential risk factors associated with development of depression in T2DM with their respective subjects’ number and/or respective odds ratio (OR) and 95% confidence interval (CI)

If necessary, we will contact the corresponding authors by email for any incomplete information and data. Finally, the extracted data will be cross-checked, a third reviewer (JRJ) will participate in discussion if there are any disagreements.

### Risk of bias assessment

Two reviewers (LG and CD) will assess the risk of bias of included studies dependently by the Agency for Healthcare Research and Quality (ARHQ) methodology checklist which is devised for cross-sectional/prevalence study quality [36]. ARHQ methodology checklist consists of 11 items, and each item will be answered with “Yes,” “No,” or “Unclear.” An item will be scored “1” if answered with “Yes” and scored “0” if answered with “Unclear” or “No.” A study will be rated as high, moderate, and low risk of bias when quality is scored 0–3, 4–6, and 7–8 respectively. Assessment results will be cross-checked, and disagreement will be determined by a third reviewer (JL).

### Data analysis

The data from each study (e.g., characteristics, context, exposures, outcomes, and findings) will be used to build evidence tables of an overall description of included studies. We will conduct random effects meta-analysis to summarize the crude prevalence, and present along with 95% confidence interval (CI). If possible, we will use odds ratio (OR) for categorical data (e.g., female and male, smoker and non-smoker) to explore potential risk factors, and it will be considered statistically significant when OR value and 95% CI is not equal to 1 and *P* < 0.05. Forest plots and tables will be used to present the results and visualize the level of heterogeneity among studies. The statistical heterogeneity among studies will be assessed by the Cochran’s *Q* test and the *I*^*2*^ statistic. *P* value < 0.10 for the *Q* test and an *I*^*2*^ > 50% will be set as the threshold for statistically significant heterogeneity. We will assess inter-rater agreement between reviewers for study inclusion, data extraction, and study quality assessment using Kappa statistics. Level of agreement will be divided into 3 categories according to Kappa statistics: fair (0.40–0.59), good (0.60–0.74), and excellent agreement (≥ 0.75) [37]. All statistical analyses will be performed using R software (vision 3.6.1) and STATA (vision 12.0) software.

### Additional analyses

We plan to conduct subgroup analyses according to gender, age, complications, and lifestyles (e.g., smoking, drinking, and exercise), different ways of identifying depression (e.g., Patient Health Questionnaire-9, Hamilton Depression Scale, or Self-rating depression scale) and other characteristics (based on cross-sectional study report). Sensitivity analysis will be performed in the following approaches: (1) excluding studies one by one to investigate the robustness of the results; (2) pooling studies’ prevalence with low, moderate, and high risk of bias respectively to explore the impact of studies’ quality on results; (3) if funnel plots is asymmetric, we will use trim and fill method to quantify the impact of publication bias on result. When there is a statistically significant heterogeneity, we will conduct meta-regression analysis to explore the sources of heterogeneity using covariates (e.g., different instruments of identifying depression, publication year, year of study).

### Meta-biases

If there are ≥10 studies in each outcome, we will use Funnel plots and Egger’s test to assess publication bias, and *P* values < 0.10 indicate potential publication bias.

## Discussion

In China, the ever-increasing prevalence of T2DM brings a great challenge for public health [5]. According to the results of cross-section studies, the prevalence of T2DM patients with depression in China ranges from 10 to 50% [25–29]. Study has shown that an early diabetes self-management education can improve depressive status, resulting in better blood glucose control in patients with newly diagnosed T2DM [38]. In addition, Pouwer F et al. [39] inferred depression screening and subsequent intensive depression management might be beneficial for T2DM-related outcomes. Therefore, detection and treatment of depression as early as possible are crucial for T2DM population. Our SR will provide a pooled prevalence of depression in Chinese T2DM patients by quantitative analysis, expecting to provide evidence for healthcare practitioners and policy makers to comprehensively consider the psychosomatic symptoms in the diagnosis and treatment of T2DM. And through exploring the potential risk factors associated with depression in T2DM, the high-risk population of depression might benefit from early interventions. To sum up, our data will highlight the need and importance of early detection and intervention for depression in patients with T2DM.

Potential limitations could be various identification scales of depression in cross-sectional studies may have impact on the accuracy of prevalence. In addition, our studies will explore the potential risk factors, and the causation between T2DM and depression cannot be identified. Nevertheless, we expect that the results of this study will draw attention to psychological health of Chinese patients with T2DM. Any amendments made to this protocol when conducting the review will be outlined in PROSPERO and reported in the final manuscript. Results will be disseminated through conference presentations and publication in a peer-reviewed journal.

## Supplementary Information

**Additional file 1.** PRISMA-P 2015 Checklist.

**Additional file 2.** Draft of Search Strategy for MEDLINE.

## Data Availability

Not applicable.

## Abbreviations

T2DM: Type 2 diabetes mellitus
IDF: International diabetes federation
PRISMA-P: Preferred Reporting Item for Systematic Review and Meta-analysis-Protocol
MOOSE: Meta-analysis Of Observational Studies in Epidemiology
PROSPERO: Prospective Register of Systematic Reviews
MeSH: Medical Subject Headings
CBM: Chinese Biomedical Literature Database
CNKI: China National Knowledge Infrastructure
VIP: Chinese Science and Technology Periodical Database
