# Supplementary material for: Prevalence and risk factors of depression in Chinese patients with type 2 diabetes mellitus: a protocol of systematic review and meta-analysis
Source: Syst Rev. 2021 Nov 25;10:302. doi: 10.1186/s13643-021-01855-7 (PMC8620640; doi:10.1186/s13643-021-01855-7)
Supplement: Supplementary file 2 — Additional file 2. Draft of Search Strategy for MEDLINE. [file 13643_2021_1855_MOESM2_ESM.docx]

**Additional file 2.** Draft of Search Strategy for MEDLINE

Search terms:

1. exp Diabetes Mellitus/
2. diabet$.tw,ot.
3. (IDDM or NIDDM or MODY or T2DM or T2D).tw,ot.

4. (non insulin$ depend$ or non insulin$ depend$ or non insulin?depend$ or non insulin?depend$).tw,ot.

5. (insulin$ depend$ or insulin?depend$).tw,ot.

6. 1 or 2 or 3 or 4 or 5

7. (depress$ or dysthymi$ or mood or affective disorder* or affective symptom*).tw,ot.

8. ((mental* or psychiatr*) adj1 (ill* or disorder* or health or disease or diagnos* or condition)).tw,ot.

9. exp DEPRESSIVE DISORDER/

10. exp Depressive Disorder, Major/

11. exp DEPRESSION/

12. 7 or 8 or 9 or 10 or 11

13. exp China/

14. (China or Chinese).af.

15. 13 or 14

16. 6 and 12 and 15
